# Betaine inhibits vascularization via suppression of Akt in the retinas of streptozotocin-induced hyperglycemic rats

**DOI:** 10.3892/mmr.2015.3613

**Published:** 2015-04-15

**Authors:** YOUNG-GIUN KIM, HYUNG-HO LIM, SUH-HA LEE, MAL-SOON SHIN, CHANG-JU KIM, HYEON JEONG YANG

**Affiliations:** 1 Department of Oriental Medical Rehabilitation, Gil Oriental Medical Hospital, College of Oriental Medicine, Gachon University, Incheon 405-760, Republic of Korea; 2 Department of Physiology, College of Medicine, Kyung Hee University, Seoul, Gyeonggi-do 130-701, Republic of Korea; 3 Department of Anesthesiology and Pain Medicine, CHA Bundang Medical Center, CHA University, Seongnam, Gyeonggi-do 463-721, Republic of Korea

**Keywords:** betaine, streptozotocin, diabetic rats, vascular endothelial growth factor, hypoxia-inducible factor-1α, Akt

## Abstract

Diabetic retinopathy is a severe microvascular complication amongst patients with diabetes, and is the primary cause of visual loss through neovascularization. Betaine is one of the components of *Fructus Lycii*. In the present study, the effects of betaine on the expression levels of vascular endothelial growth factor (VEGF) and hypoxia-inducible factor (HIF)-1α in association with the Akt pathway were investigated in the retinas of streptozotocin (STZ)-induced diabetic rats using western blot and immunohistochemical analyses. The results of the present study revealed that the expression levels of VEGF, HIF-1α, and Akt were increased in the retinas of the STZ-induced diabetic rats. Betaine treatment attenuated this increase in VEGF and HIF-1α expression via suppression of diabetes-induced Akt activation in the retinas of the diabetic rats. The results suggested that betaine may potentially be used to delay the onset of complications associated with diabetic retinopathy via inhibition of retinal neovascularization in patients with diabetes.

## Introduction

Diabetes mellitus is a severe metabolic disease, and numerous complications are associated with the characteristic hyperglycemia of this disease (1–3). Of these, diabetic retinopathy is one of the major microvascular complications amongst diabetic patients, and is the primary cause of visual loss through neovascularization (1).

Vascular endothelial growth factor (VEGF) is a potent angiogenic and vascular hyperpermeability factor, and has a key role in the pathogenesis underlying diabetic retinopathy (2). VEGF is produced by multiple types of cell, including retinal pigment epithelium, ganglion cells, Müller cells, pericytes and the smooth muscle cells of the human retina and choroid, and is mainly modulated by tissue oxygen content (3). The transcriptional regulation of VEGF is mediated by hypoxia-inducible factor (HIF)-1 (4).

HIF-1 is the primary hypoxic signaling protein in cells for regulating angiogenesis, and is a transcription factor that is regarded as a ‘master switch’, responsible for the regulation of all oxygen-dependent retinal diseases (5). Downregulation of HIF-1 inhibits neovascularization in proliferative diabetic retinopathy (4). HIF-1 is a heterodimeric transcriptional factor composed of a labile α subunit (120 kDa) and a stable β subunit (92 kDa) (5). HIF-1α activity is regulated by posttranslational modification-associated processes, whereas HIF-1β is constitutively expressed (6). HIF-1 induces the transcription of genes whose protein products function to enhance O_2_ delivery, for example erythropoietin and VEGF, which stimulate erythropoiesis and angiogenesis, respectively; or to induce metabolic adaptations to facilitate function under reduced O_2_ conditions, including glucose transporters and glycolytic enzymes (5). HIF-1α promotes neovascularization (7,8), by increasing VEGF expression (9).

Akt is a member of a class of serine or threonine protein kinases, and is a major effector in the phosphoinositide 3-kinase (PI3K) signaling pathway (10,11). Akt has a significant role in multiple cellular processes, including cell survival, metabolism, growth, proliferation and mobility (10), and additionally regulates vascular homeostasis and angiogenesis (11). Growth factors, cytokines and other signaling molecules stimulate HIF-1α protein synthesis via activation of the PI3K/Akt signaling pathways (10,11). Phosphorylated (p)Akt denotes activated Akt, and the PI3K/Akt pathway is involved in modulating the expression of HIF1-α and VEGF (9).

Betaine is an alkaloid, which is occasionally classified as an amino acid, that is found in capsicum, silybum (the source of liver-protective flavonoid, silymarin) and *Beta vulgaris* (12,13). Betaine is found amongst various animals, plants and microorganisms, and dietary sources rich in betaine include seafood, in particular marine invertebrates, wheat germ or wheat bran and spinach. The primary physiological role of betaine is as an osmolyte and methyl donor for transmethylation. In its capacity as an osmolyte, betaine protects cells, proteins and enzymes against environmental stressors. In addition, betaine is a crucial nutrient required for the prevention of chronic diseases (12). Betaine is a component of *Fructus lycii*, and is known to enhance visual acuity (13). However, the effect of betaine on the neovascularization of the retinas of patients with diabetes, which is the underlying cause of diabetic retinopathy, has remained to be elucidated.

In the present study, the effect of betaine on the expression of VEGF and HIF-1α in association with Akt activation in the retinas of streptozotocin (STZ)-induced diabetic rats was investigated using western blot analysis and immunohistochemistry.

## Materials and methods

### Animals and treatments

Forty male Sprague-Dawley rats (Dae Han Bio Link Co., Ltd., Seoul, Korea) weighing 120±10 g (aged five weeks) were used in the present study. The rats were housed under controlled temperature (20±2°C) and lighting conditions (07:00 to 19:00 h), with *ad libitum* access to food and water throughout the experimental period. The experimental procedures were performed in accordance with the animal care guidelines of the National Institutes of Health and the Korean Academy of Medical Sciences (Seoul, Korea), and the study was approved by the Kyung Hee Insitutional Animal Care and Use Committee (Seoul, Korea). The animals were randomly divided into four groups (n=10 per group): The control group, the STZ-induced diabetes group, STZ-induced diabetes and 250 mg/kg betaine-treated group and the STZ-induced diabetes and 500 mg/kg betaine-treated group. The control group received the same volume of water for the same duration. Betaine was purchased from Sigma-Aldrich (St. Louis, MO, USA). Four weeks following STZ administration, betaine was orally administrated to the rats once a day for 14 consecutive days at the respective doses for each group.

### Induction of diabetes

Diabetes was induced in the experimental animals with a single intraperitioneal (i.p.) injection of STZ (60 mg/kg, dissolved in 10 mM citrate buffer; pH 4.5; Sigma-Aldrich) administered to each animal. Blood glucose levels were determined two days after STZ injection using a blood glucose tester (Arkray, Kyoto, Japan). Only those rats with blood glucose levels of ≥300 mg/dl were confirmed to have diabetes and used in the diabetes groups. Subsequently, blood glucose levels were measured at 0, 2, 4 and 6 weeks following commencement of the experiment.

### Tissue preparation

The animals were anesthetized using Zoletil 50^®^ (10 mg/kg, i.p.; Vibac Laboratories, Carros, France), transcardially perfused with 50 mM phosphate-buffered saline and fixed with a freshly prepared solution of 4% paraformaldehyde (Sigma-Aldrich) in 100 mM phosphate buffer (pH 7.4; Sigma-Aldrich). The retinas were dissected and postfixed overnight in 4% paraformaldehyde with 100 mM phosphate buffer, and then transferred into a 30% sucrose solution (Sigma-Aldrich) for cryoprotection. A freezing microtome (CM 1510-3; Leica Microsystems GmbH, Nussloch, Germany). was used to slice 20-*µ*m coronal sections of the retinas.

### Western blot analysis of VEGF, HIF1-α and pAkt expression

Western blot analyses were conducted according to a previously described method (14,15). Retinal tissues were lysed in ice-cold whole cell lysate buffer, which comprised 50 mM HEPES (pH 7.5), 150 mM NaCl, 10% glycerol, 1% Triton X-100, 1.5 mM magnesium chloride hexahydrate, 1 mM ethyleneglycol-bis-(β-aminoethyl ether)-*N,N*′-tetraacetic acid, 1 mM phenylmethylsulfonyl fluoride (PMSF), 2 *µ*g/ml leupeptin, 1 *µ*g/ml pepstatin, 1 mM sodium orthovanadate and 100 mM sodium fluoride. Additionally, rat retina tissues were lysed in a lysis buffer containing 50 mM Tris-HCl (pH 7.5), 150 mM NaCl, 0.5% deoxycholic acid, 1% Nonidet P40, 0.1% SDS, 1 mM PMSF and 100 mg/ml leupeptin. The mixture was incubated at 4°C for 30 min. Cell debris was removed by microcentrifugation at 19,000 x g for 20 min at 4°C, followed by snap freezing of the supernatant. The protein concentration was measured using a Bio-Rad colorimetric protein assay kit (Bio-Rad Laboratories, Inc., Hercules, CA, USA). Protein (30 *µ*g) was separated on 12% SDS-PAGE and transferred onto a nitrocellulose membrane (Schleicher & Schuell GmbH, Dassel, Germany). The membranes were incubated with the following primary antibodies: Mouse monoclonal anti-actin antibody (cat. no. sc-8432), rabbit polyclonal anti-HIF-1α antibody (cat. no. sc-10790) and mouse monoclonal anti-VEGF antibody (cat. no. sc-7269) (1:1,000; Santa Cruz Biotechnology Inc., Dallas, TX, USA); rabbit polyclonal anti-Akt antibody (cat. no. 9272) and rabbit monoclonal anti-pAkt antibody (cat. no. 4060) (1:1,000; Cell Signaling Technology Inc., Beverly, MA, USA) at 4°C overnight. The membranes were then incubated with anti-mouse (1:2,000; cat. no. RPN4201; Amersham Pharmacia Biotechnology GmbH, Freiburg, Germany) and anti-rabbit (1:2,000; cat. no. sc-2054; Santa Cruz Biotechnology, Inc.) antibodies at room temperature for 1 h. Protein bands were detected using an enhanced chemiluminescence detection system (Santa Cruz Biotechnology, Inc.).

### Immunohistochemical analysis of VEGF, HIF1-α and pAkt expression

Immunohistochemical analyses were conducted as previously described (16,17). The frozen retinal sections were incubated overnight at 4°C with mouse monoclonal anti-VEGF antibody (1:200; cat. no. sc-7269; Santa Cruz Biotechnology, Inc.), rabbit polyclonal anti-HIF1-α antibody (1:500; cat. no. sc-10790; Santa Cruz Biotechnology, Inc.) and rabbit monoclonal anti-pAkt antibody (1:200; cat. no. 4060; Cell Signaling Technology, Inc.). Subsequently, the sections were incubated for a further 1 h at room temperature with biotinylated anti-mouse secondary antibody (1:200; cat. no. BA2000; Vector Laboratories, Inc., Burlingame, CA, USA) and biotinylated anti-rabbit secondary antibody (1:200; cat. no. BA1000; Vector Laboratories, Inc.). The bound secondary antibody was then amplified using a Vector Elite ABC kit^®^ (1:100; Vector Laboratories, Inc.). The antibody-biotin-avidin-peroxidase complexes were visualized using 0.03% 3,3′diaminobenzidine (Sigma-Aldrich) and the sections were mounted onto gelatin-coated slides (Marienfeld-Superior, Lauda-Königshofen, Germany). The slides were air dried at room temperature overnight, and cover-slips were subsequently mounted with Permount^®^ (Thermo Fisher Scientific, Waltham, MA, USA).

### Statistical analysis

Following staining, the number of immunoreactive cells per 1,000-*µ*m length of retinal section were counted for each rat. Results were analyzed using one-way analysis of variance followed by Duncan’s post-hoc test, and values are presented as the mean ± standard error of the mean. Statistical analyses were conducted using SPSS version 21.0 (IBM, Armonk, NY, USA). P<0.05 was considered to indicate a statistically significant difference between values.

## Results

### Effect of betaine on blood glucose levels

At 0, 2, 4 and 6 weeks of the experiment, blood glucose levels were 134.08±0.84, 134.57±4.67, 137.29±12.14 and 137.79±9.45 mg/dl, respectively, in the control group; 134.08±0.84, 414.14±9.75, 458.00±40.01 and 457.14±19.16 mg/dl, respectively, in the STZ-induced diabetes group; 134.08±0.84, 419.13±12.53, 460.75±35.84 and 405.75±19.88 mg/dl, respectively, in the STZ-induced diabetes and 250 mg/kg betaine-treated group and 134.08±0.84, 419.00±13.22, 453.37±35.36 and 391.75±22.75 mg/dl, respectively, in the STZ-induced diabetes and 500 mg/kg betaine-treated group. Blood glucose levels were significantly increased following STZ injection (P<0.05). Two weeks of betaine treatment decreased blood glucose level in the diabetic rats, however this decrease was not statistically significant (P>0.05).

### Betaine attenuates the STZ-induced increase in VEGF expres- sion in the retina

**Figure 1:**
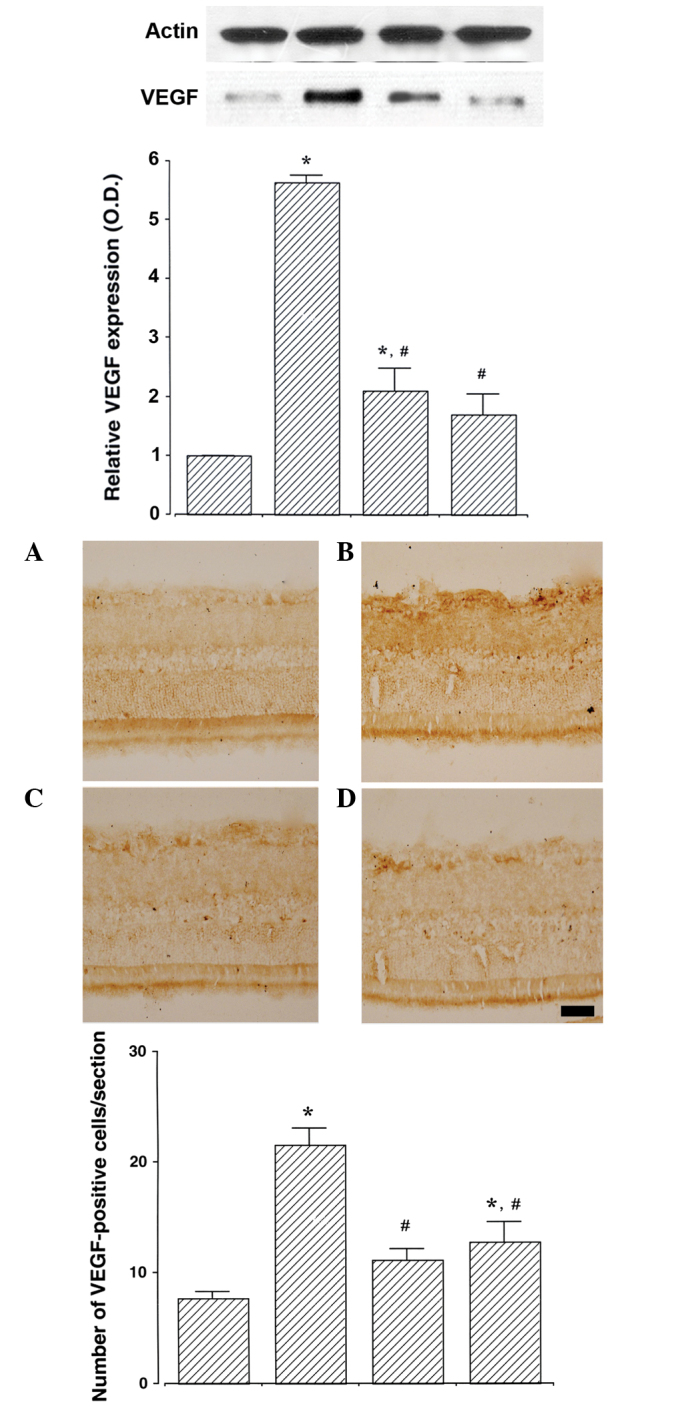
Effect of betaine on VEGF expression in the retina. Upper: Western blot analysis of VEGF expression. Actin was used as the internal control. Lower: Immunohistochemical analysis of VEGF expression. Scale bar, 25 mm. Values are presented as the mean ± standard error of the mean. (A) Control group, (B) STZ-induced diabetes group, (C) STZ-induced diabetes and 250 mg/kg betaine-treated group and (D) STZ-induced diabetes and 500 mg/kg betaine-treated group. ^*^P<0.05 compared with the control group, ^#^P<0.05 compared with the STZ-induced diabetes group. VEGF, vascular endothelial growth factor; STZ, streptozotocin; O.D. optical density.

The expression of VEGF in the retinas of the control group was set as 1.0. The expression of VEGF was 5.64±0.12 in the STZ-induced diabetes group, 2.11±0.37 in the STZ-induced diabetes and 250 mg/kg betaine-treated group and 1.69±0.35 in the STZ-induced diabetes and 500 mg/kg betaine-treated group. STZ-induced diabetic rats demonstrated enhanced VEGF expression in the retina (P<0.05) compared with that of the control group. By contrast, betaine treatment suppressed VEGF expression in the retinas of the diabetic rats (P<0.05), compared with that of the STZ-induced diabetes group. A higher dose of betaine exerted a more potent suppressive effect on VEGF expression levels (Fig. 1, upper panel).

The number of VEGF-positive cells in the retinas was 7.70±0.62/section in the control group, 21.48±1.56/section in the STZ-induced diabetes group, 11.09±0.99/section in the STZ-induced diabetes and 250 mg/kg betaine-treated group and 12.75±1.79/section in the STZ-induced diabetes and 500 mg/kg betaine-treated group. An increased number of VEGF-positive cells were detected in the retinas of STZ-induced diabetes rats compared with those of the control group (P<0.05). By contrast, betaine treatment inhibited this increase in the number of VEGF-positive cells in the retinas of the diabetic rats (P<0.05). A lower dose of betaine exerted a more potent inhibitory effect on the number VEGF-positive cells (Fig. 1, lower panel).

### Betaine attenuates the STZ-induced increase in HIF-1α expression in the retina

**Figure 2:**
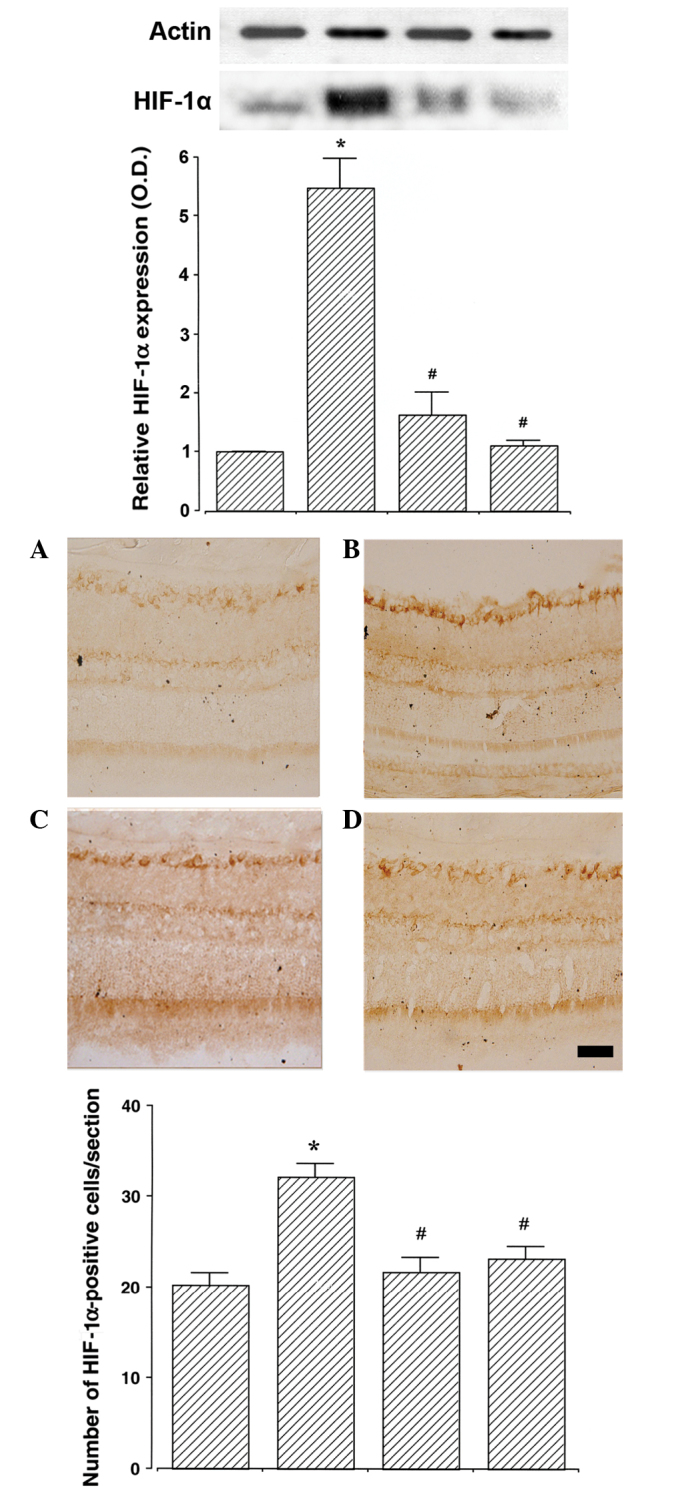
Effect of betaine on HIF-1α expression in the retina. Upper: Western blot analysis of HIF-1α expression. Actin was used as the internal control. Lower: Immunohistochemical analysis of HIF-1α expression. Scale bar, 25 mm. Values are presented as the mean ± standard error of the mean. (A) Control group, (B) STZ-induced diabetes group, (C) STZ-induced diabetes and 250 mg/kg of betaine-treated group and (D) STZ-induced diabetes and 500 mg/kg betaine-treated group. ^*^P<0.05 compared with the control group. ^#^P<0.05 compared with the STZ-induced diabetes group. STZ, streptozotocin; HIF-1α, hypoxia inducible factor-1α.

The expression of HIF-1α in the retinas of the control group was set as 1.0. The expression of HIF-1α was 5.48±0.51 in the STZ-induced diabetes group, 1.64±0.38 in the STZ-induced diabetes and 250 mg/kg betaine-treated group and 1.11±0.10 in the STZ-induced diabetes and 500 mg/kg betaine-treated group. STZ-induced diabetic rats demonstrated enhanced levels of HIF-1α expression in the retina compared with those of the control group (P<0.05). Conversely, betaine treatment suppressed HIF-1α expression in the retinas of the STZ-induced diabetes rats (P<0.05). Higher doses of betaine exerted a more potent suppressive effect on the HIF-1α expression, although this effect was not significant (Fig. 2, upper).

The number of HIF-1α-positive cells in the retinas was 20.26±1.29/section in the control group, 32.16±1.49/section in the STZ-induced diabetes group, 21.73±1.56/section in the STZ-induced diabetes and 250 mg/kg betaine-treated group and 23.19±1.31/section in the STZ-induced diabetes and 500 mg/kg betaine-treated group. STZ-induced diabetes rats exhibited an increased number of HIF-1α-positive cells in the retinas, compared with those of the control group (P<0.05). By contrast, betaine treatment inhibited this increase in the number of HIF-1α-positive cells in the retinas of the diabetic rats (P<0.05; Fig. 2, lower panel).

### Betaine ameliorates the STZ-induced increase in pAkt expression in the retina

**Figure 3:**
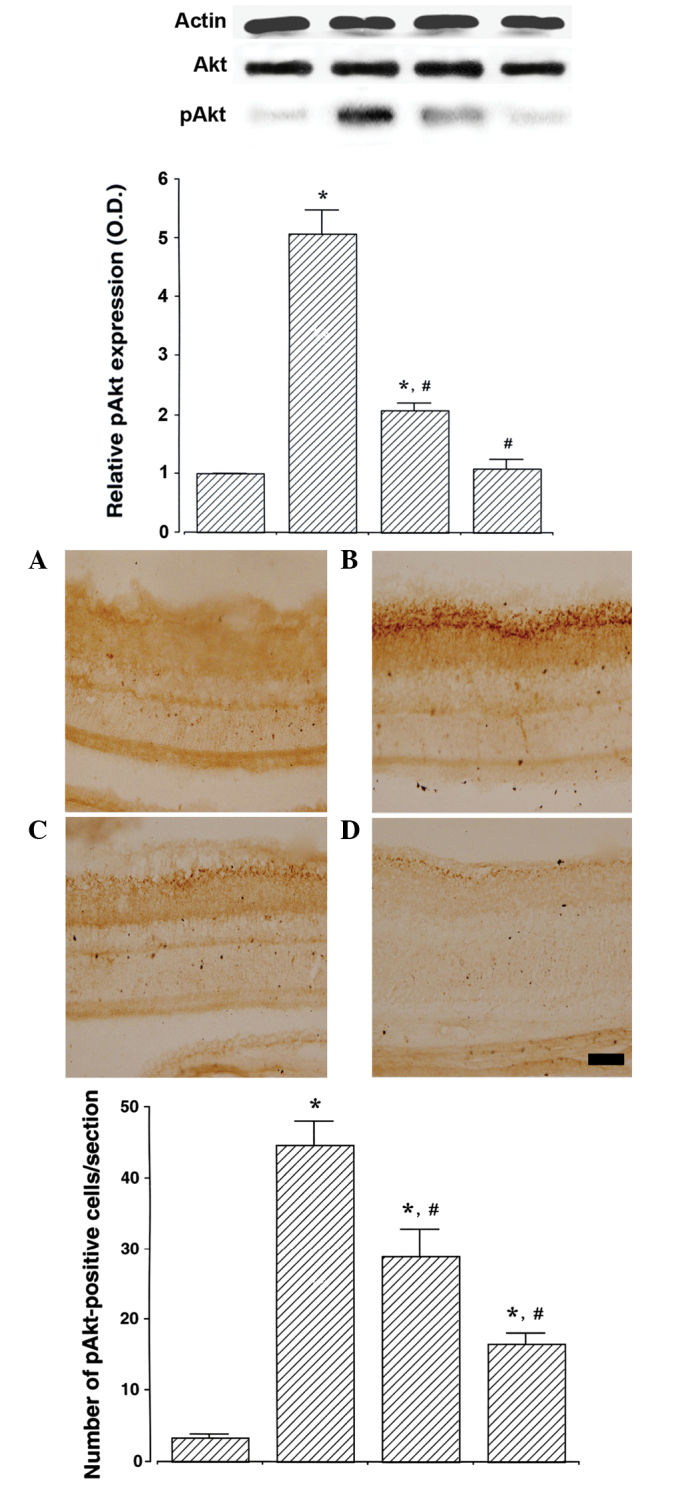
Effect of betaine on pAkt expression in the retina. Upper: Western blot analysis of pAkt expression. The expression of pAkt was compared with Akt expression. Lower: Immunohistochemical analysis of pAkt expression. Scale bar, 25mm. Values are presented as the mean ± standard error of the mean. (A) Control group, (B) STZ-induced diabetes group, (C) STZ-induced diabetes and 250 mg/kg of betaine-treated group and (D) STZ-induced diabetes and 500 mg/kg of betaine-treated group. ^*^P<0.05 compared with the control group, ^#^P<0.05 compared with the STZ-induced diabetes group. STZ, streptozotocin; pAkt, phosphorylated Akt.

The expression of pAkt in the retinas of the control group was set as 1.0. The expression of pAkt was 5.07±0.40 in the STZ-induced diabetes group, 2.08±0.13 in the STZ-induced diabetes and 250 mg/kg betaine-treated group and 1.09±0.15 in the STZ-induced diabetes and 500 mg/kg betaine-treated group. STZ-induced diabetes rats revealed enhanced pAkt expression in the retinas compared with those of the control rats (P<0.05). However, betaine treatment was able to suppress this increase in pAkt expression in the retinas of the diabetic rats (P<0.05). A higher dose of betaine exerted a more potent suppressive effect on pAkt expression (Fig. 3, upper panel).

The number of pAkt-positive cells in the retina was 3.41±0.36/section in the control group, 44.71±3.25/section in the STZ-induced diabetes group, 28.87±3.82/section in the STZ-induced diabetes and 250 mg/kg betaine-treated group and 16.63 ± 1.42/section in STZ-induced diabetes and 500 mg/kg betaine-treated group. STZ-induced diabetes resulted in an increase in the number of pAkt-positive cells in the rat retinas compared with those in the control group (P<0.05). By contrast, betaine treatment inhibited this increase in the number of pAkt-positive cells in the retinas of the diabetic rats (P<0.05). A higher dose of betaine exerted a more potent inhibitory effect on the number of pAkt-positive cells (Fig. 3, lower panel). These results suggest that betaine is able to suppress Akt activation.

## Discussion

Suppression of angiogenesis is a key therapeutic strategy in the prevention of the progression of diabetic retinopathy (18,19). Steroid dexamethasone, laser photocoagulation and vitrectomy have clinically been used for the prevention of neovascularization amongst patients with diabetes (1,18).

In the present study, the anti-angiogenic effect of betaine was evaluated using an STZ-induced hyperglycemic rat model. Betaine has previously been demonstrated to protect internal organs, reduce vascular risk factors and enhance athletic performance (12). Betaine reduces homocysteine level in homocystinuria, decreases serum homocysteine level and increases brain methionone and S-adenosylmethionine, functions which may delay the progression of Alzheimer’s disease (20).

The STZ-induced diabetic rat model is the most widely used animal model for diabetes (16,17), and is also extensively used in the study of diabetic neuropathy in particular (21,22). Vascular changes, including microaneurysms, decreased pericyte number, increased vascular permeability, breakdown of blood-retinal barrier and early changes in growth factor expression, were observed in the STZ-induced diabetic rats (23,24).

In the present study STZ-induced diabetic rats demonstrated enhanced VEGF expression in the retina, an effect which was attenuated following betaine treatment. VEGF is an endothelial angiogenic and vasopermeability factor, which induces functional changes in the retinal pigment epithelium (25). VEGF expression is upregulated by hypoxia and ischemia (26), and VEGF directly stimulates retinal neovascularization (27,28). Enhanced expression of VEGF occurs as a result of the upregulation of survivin, which is activated by the PI3K/Akt signaling pathway (29). Suppression of VEGF expression in the retina has been demonstrated to enhance vision in multiple neovascular eye diseases, including diabetic retinopathy and age-associated macular degeneration (30,31).

In the present study, STZ-induced diabetic rats additionally demonstrated enhanced HIF-1 expression in the retina. Betaine treatment suppressed this increase in HIF-1 expression in the STZ-induced diabetic rats. Previously, increased levels of HIF-1α were observed in the ischemic retinas of the retinopathy mouse model, and HIF-1α expression was correlated with VEGF expression (27). HIF-1α regulates the expression of numerous genes required for normal cellular function and survival under various stressful conditions (32). The retina is sensitive to oxygen tension, and oxygen has a key role in the stabilization of HIF-1α function. When oxygen tension is normal, HIF-1α is rapidly oxidized by hydroxylase enzymes; however, when cells enter a hypoxic state, HIF-1α degradation is inhibited, and HIF-1α activates VEGF and erythropoietin (4–6). HIF-1α is closely associated with oxygen-dependent retinal diseases, including von Hippel-Lindau, proliferative diabetic retinopathy, retinopathy of prematurity and glaucoma (4). HIF-1 mediates the role of Akt by promoting VEGF expression; therefore, promotion of the Akt-HIF-1α-VEGF signaling pathway contributes to the induction of angiogenesis (33).

The results of the present study revealed an increase in pAkt expression in the retinas of STZ-induced diabetic rats, an effect that was ameliorated by betaine treatment. VEGF-induced endothelial cell migration requires Akt activation (34). Activation of Akt signaling in the endothelial cells stimulates endothelial cell bioactivity and angiogenesis (35), and pAkt is predominantly expressed in the inner nuclear layer of the diabetic retina (36). Furthermore, the hypoxia-induced expression of HIF-1α and VEGF requires activation of the PI3K/Akt pathway (9).

The results of the present study supported the hypothesis that Akt activation is an upper signaling pathway, which triggers a proliferative response in the endothelial cells by enhancing VEGF expression, leading to the induction of neovascularization. The results also suggested that the inhibition of Akt activation may ameliorate neovascularization via suppression of VEGF expression. In addition, betaine treatment alleviated diabetes-induced vascularization by suppressing VEGF and HIF-1α expression via downregulation of pAkt expression in the retina of the STZ-induced diabetic rats. In conclusion, the results of the present study suggested a potential role for betaine in the prevention and/or delay of complications of diabetic retinopathy by inhibiting retinal neovascularization in patients with diabetes.
